# Dynamic regulation of gene expression using sucrose responsive promoters and RNA interference in *Saccharomyces cerevisiae*

**DOI:** 10.1186/s12934-015-0223-7

**Published:** 2015-04-01

**Authors:** Thomas C Williams, Monica I Espinosa, Lars K Nielsen, Claudia E Vickers

**Affiliations:** Australian Institute for Bioengineering and Nanotechnology (AIBN), The University of Queensland, St. Lucia, QLD 4072 Australia

**Keywords:** Yeast, Sucrose, GFP, Metabolic engineering, Synthetic biology, Promoter, SUC2, TEF1, Diauxic shift

## Abstract

**Background:**

Engineering dynamic, environmentally- and temporally-responsive control of gene expression is one of the principle objectives in the field of synthetic biology. Dynamic regulation is desirable because many engineered functions conflict with endogenous processes which have evolved to facilitate growth and survival, and minimising conflict between growth and production phases can improve product titres in microbial cell factories. There are a limited number of mechanisms that enable dynamic regulation in yeast, and fewer still that are appropriate for application in an industrial setting.

**Results:**

To address this problem we have identified promoters that are repressed during growth on glucose, and activated during growth on sucrose. Catabolite repression and preferential glucose utilisation allows active growth on glucose before switching to production on sucrose. Using sucrose as an activator of gene expression circumvents the need for expensive inducer compounds and enables gene expression to be triggered during growth on a fermentable, high energy-yield carbon source. The ability to fine-tune the timing and population density at which gene expression is activated from the *SUC2* promoter was demonstrated by varying the ratio of glucose to sucrose in the growth medium. Finally, we demonstrated that the system could also be used to repress gene expression (a process also required for many engineering projects). We used the glucose/sucrose system to control a heterologous RNA interference module and dynamically repress the expression of a constitutively regulated GFP gene.

**Conclusions:**

The low noise levels and high dynamic range of the *SUC2* promoter make it a promising option for implementing dynamic regulation in yeast. The capacity to repress gene expression using RNA interference makes the system highly versatile, with great potential for metabolic engineering applications.

**Electronic supplementary material:**

The online version of this article (doi:10.1186/s12934-015-0223-7) contains supplementary material, which is available to authorized users.

## Background

Microorganisms possess a variety of mechanisms for modulating gene expression levels according to changes in their environment. The ability to engineer dynamic regulation is highly desirable in microbial cell factories because many of the most productive genetic modifications used for optimising engineering objectives are also detrimental to cell growth and survival. Engineered pathways compete for the carbon flux, redox potential, and ATP required by the cell for normal growth [1]. Engineered pathways can also result in accumulation of intermediates, side-products or end-products that are highly toxic to the host cell [2]. If a production strain cannot reach a high population density due to the metabolic burden imposed by an engineered pathway, then product titers are inherently limited. For genetic modifications that impose the most severe limits on growth, it is essential that they be implemented near or after the completion of a growth phase. One of the most promising techniques to optimise cell factory performance is to control the expression/repression of relevant genes using dynamic regulation.

*S. cerevisiae* (yeast) is an industrial microorganism which has been employed for the production of fuels, chemicals, and pharmaceuticals [3]. There are a limited number of molecular tools available for implementing dynamic regulation in yeast, with most being inadequate for industrial application. For example, the commonly used galactose induction system [4] has a noise level (expression in the absence of inducer) which is unacceptable for some applications such as controlling RNA interference [5]. Furthermore, galactose is prohibitively expensive for use at an industrial scale [6]. Previous efforts to implement dynamic regulation under industrially relevant conditions required the knockout of galactose utilization genes so that a small amount of galactose can be added to fermentations as a gratuitous inducer for gene expression [6]. High concentrations of fermentable carbon sources repress gene expression from galactose promoters (GAL) via a carbon catabolite repression mechanism [7], and galactose utilisation genes are not activated during growth on sucrose [8]. This means that cheap, fermentable sugars such as glucose or sucrose cannot be used during a production phase when the GAL promoters are used to achieve dynamic regulation. To circumvent this limitation, ethanol has been used as a carbon source for fed-batch cultures with the GAL promoters [6]. However, this is also problematic because ethanol is more expensive than commonly used sugar feedstocks, and is in fact a common commercial product of industrial yeast fermentations. Other induction systems such as the doxycycline inducible promoters have the advantage of being completely orthologous to native yeast regulation [9], but are also too expensive to be employed at a large scale. There are many other carbon source regulated promoters in yeast that could potentially be used for dynamic regulation [10], including the *ADH2* promoter that is activated during growth on ethanol, the low-oxygen regulated *DAN1* promoter, and the low phosphate activated *PHO5* promoter [11]. These options all rely on the absence of a growth component that enables maximal metabolic flux and gene expression capacity (such as glucose or sucrose). There is therefore a significant unmet need for control systems that enable dynamic regulation and have suitable properties for industrial use of cell factories such as yeast. An ideal promoter would have low noise, high dynamic range, switch-like activation, high absolute expression levels, sustained induction, and high activity on carbon sources that support high metabolic flux.

To expand the toolbox of dynamic regulatory systems in yeast we have explored the use of promoters regulated by glucose de-repression in the presence of sucrose. Sucrose is a preferred feedstock for industrial scale fermentations due to sugarcane being a more environmentally friendly source of sugar than glucose which has been derived from corn [12,13]. Promoters that can regulate high levels of gene expression during a sucrose-fed production phase are therefore highly desirable. We sought to identify and characterise the spatiotemporal expression dynamics of promoters which are repressed during growth on glucose, and up-regulated during growth on sucrose. We applied these promoters to dynamic over-expression, and also to dynamic repression via a heterologous RNA interference module that can be used to destroy target mRNA according to base pair complementarity of expressed antisense RNA [5].

## Results and discussion

### Screening for sucrose responsive promoters

The most important properties to consider when assessing mechanisms for dynamic regulation are the dynamic range (induced minus non-induced expression levels) and noise levels (non-induced minus background). Four different promoters were screened with the aim of achieving low expression levels in the presence of glucose, and high expression in the presence of sucrose. Many promoters are known to be de-repressed in the presence of low glucose in yeast [10], little or no published data is available describing how these promoters behave on sucrose as the sole carbon source. It therefore could not be assumed that promoters known to be de-repressed by low glucose would be active on sucrose. For metabolic engineering applications in yeast it is desirable to have a production phase with a cheap carbon source. Promoters which are not only de-repressed by low glucose, but that are activated during growth on the cheap industrial carbon source sucrose are therefore highly desirable for industrial yeast fermentations.

Four promoters were selected based on literature which suggested they may be differentially regulated by sucrose. SUC2p is an invertase enzyme that hydrolyses sucrose into glucose and fructose; expression of SUC2p is strongly repressed by glucose [10,14]. Transcription of *SUC2* is repressed around 200-fold when glucose levels are high and is activated only after glucose levels fall below 0.1% w/v [15-18]. The *MAL12* gene encodes a disaccharide transporter which is highly induced in the presence of sucrose in non-laboratory strains of *S. cerevisiae* [19,20]. Glc3p is a glycogen branching enzyme essential for glycogen accumulation, and the Gph1p enzyme breaks down glycogen during stationary phase [21-23]. Both *GLC3* and *GPH1* transcripts have been observed to be up-regulated during growth on sucrose [24].

**Figure 1:**
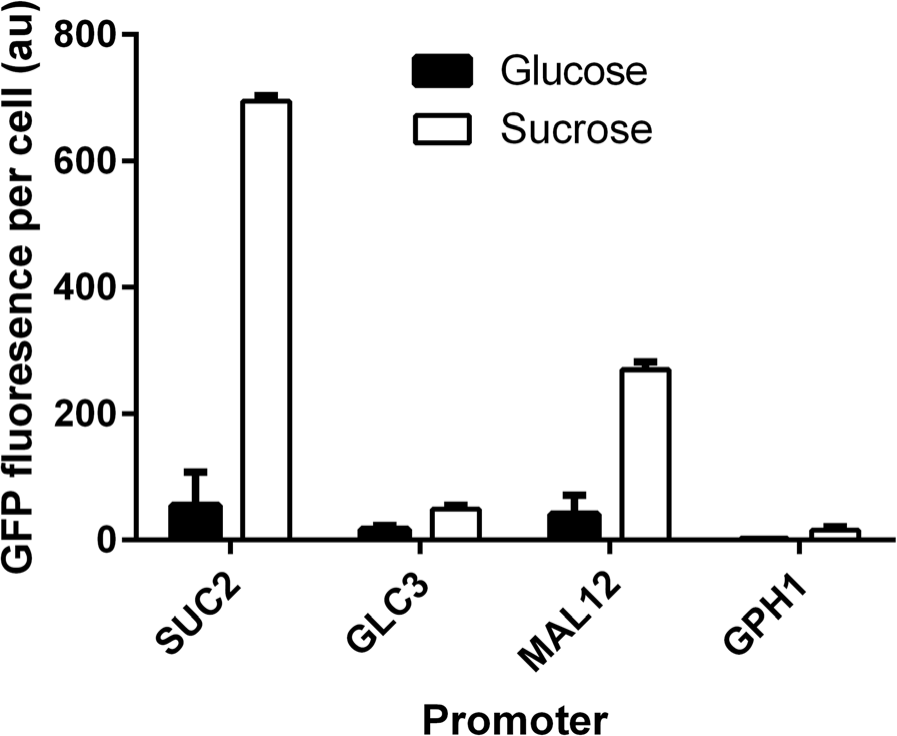
**Testing promoters for differential expression on sucrose.** *SUC2, GLC3, MAL12*, and *GPH1* promoters were used to control GFP expression. Each strain was grown to mid-exponential phase on minimal medium containing 10 g/L glucose or 10 g/L sucrose prior to flow cytometry-based GFP measurement. Mean GFP fluorescence levels in arbitrary units (au) from duplicate fermentations with error bars representing the standard deviation are shown.

Yeast strains were generated with promoter-GFP fusion constructs for the promoters *P*_*SUC2*_, *P*_*MAL12*_, *P*_*GLC3*_, or *P*_*GPH1*_ integrated into the genome at the *URA3* locus in single copy. Each strain was grown in glucose- or sucrose-containing medium with GFP fluorescence measured in the mid exponential phase (OD_660nm_ of ~1.5) after 8 hours of growth (Figure 1).

Each promoter had a significantly higher expression level on sucrose compared to on glucose. The *SUC2* promoter had both the highest dynamic range (12.5-fold induction), and the highest absolute expression level. These properties made the *SUC2* promoter an attractive choice for sucrose-mediated dynamic regulation in *S. cerevisiae*. Although this experiment was successful in identifying the best promoter, it only provided a ‘snapshot’ of expression levels during the exponential growth phase. In order to gain a deeper understanding of noise levels and dynamic range for comparison with other published data it is necessary to understand the spatiotemporal dynamics of gene expression throughout a fermentation.

### Fine-tuning temporal gene expression dynamics using different glucose:sucrose ratios

**Figure 2:**
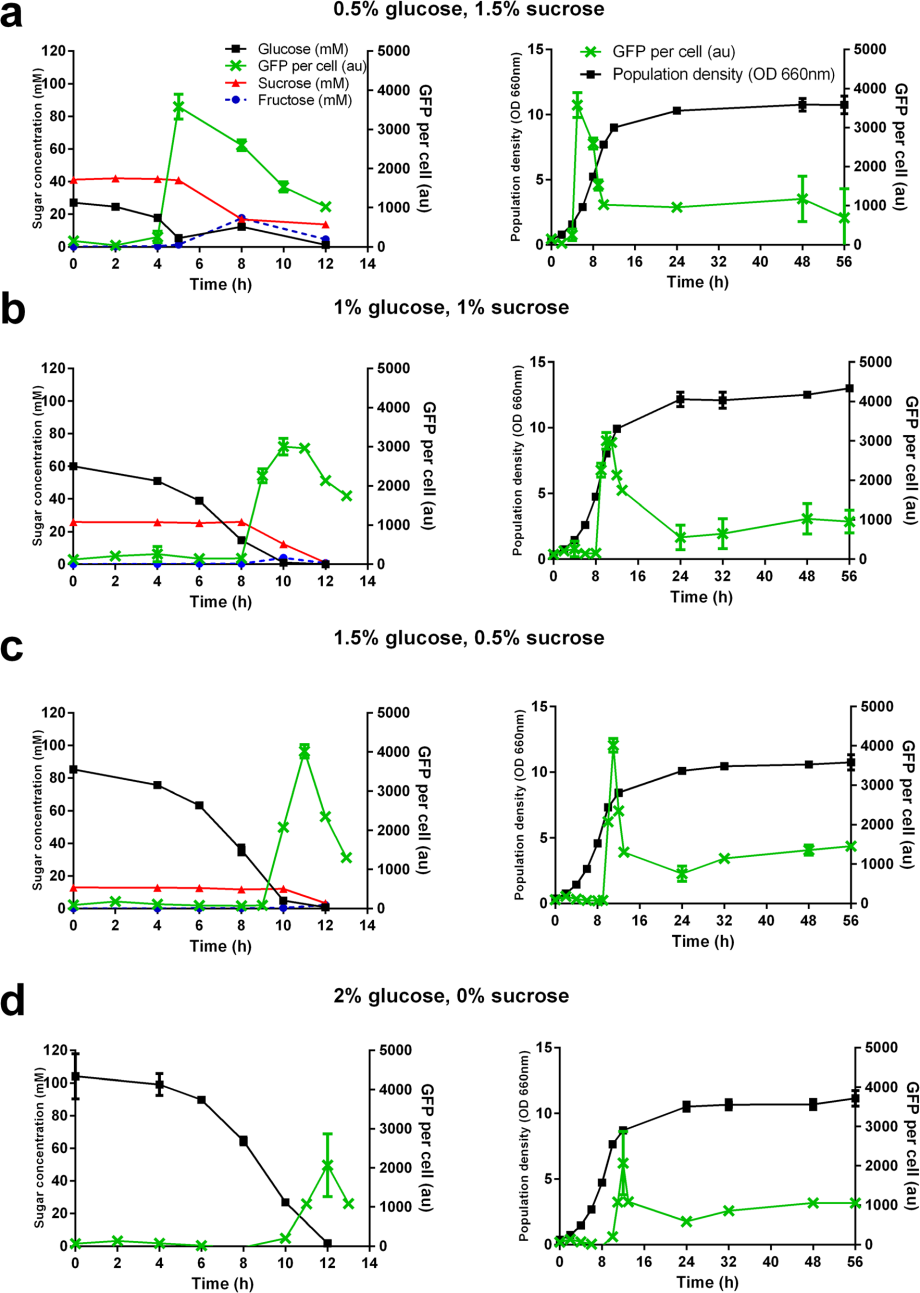
**Tuning the timing of gene expression using different ratios of glucose to sucrose.** A strain expressing the destabilized GFP gene driven by the *SUC2* promoter was grown in media containing the indicated concentrations of glucose and sucrose. Extracellular glucose, sucrose, and fructose concentrations were measured using HPLC during the initial growth phase alongside GFP expression levels. Population density (OD_660nm_) and GFP expression levels were measured up to 56 hours of shake-flask cultivation. All data points and error bars represent the mean and standard deviation from triplicate cultivations.

Implementation of sucrose-mediated dynamic regulation would involve use of a growth medium which contains a known ratio of glucose to sucrose. In theory this would result in *P*_*SUC2*_-regulated genes being tightly repressed during a growth phase where glucose is preferentially consumed, then switched ‘on’ during a sucrose-consuming phase. When growth media with different ratios of glucose to sucrose were used to grow a *P*_*SUC2*_*-GFP* expressing strain, the timing of GFP induction responded according to glucose concentration (Figure 2a-d). A destabilized version of the GFP gene with a half-life of ~20 minutes (yEGFPCLN2PEST [25]) was used so that any decreases in gene expression level would not be obscured by the high stability (half-life of ~ 7 hours) of the regular yEGFP gene.

In addition to the fine-tuning of gene expression, the time-course analysis of *SUC2* promoter activity was necessary to gain more accurate insight into expression levels and dynamic range. As expected, GFP expression from the *SUC2* promoter was strongly repressed during growth on glucose under all conditions. GFP expression levels during the glucose consumption phase were usually indistinguishable from autofluorescence, indicating tight regulation and a lack of undesirable leakiness. Once glucose concentrations dropped below about 5 mM, there was a rapid induction of GFP expression as the *SUC2* promoter was activated. GFP levels from the *SUC2* promoter peaked immediately after the switch from glucose to sucrose at around 4000 au before decreasing to 700–1000 au (Figure 2). This is consistent with the *SUC2* expression level and dynamic range seen in the promoter screen, where populations had been growing on sucrose-only medium for 8 hours and would have progressed past the initial induction phase (Figure 1). As previously reported, the *SUC2* promoter was activated after glucose was depleted even in the absence of sucrose [26]. When sucrose was present in the growth medium (Figure 2a-c) there was a 2 fold higher GFP expression level (~4000 au) (Figure 2a-c) compared to when sucrose was absent (~2000 au) (Figure 2d). As far as we are aware there is no known regulatory mechanism that can account for the increased *SUC2* mediated gene expression in the presence of sucrose compared to in the absence of glucose (during the diauxic shift). It is possible that the phenomenon shown in Figure 2d could simply reflect a greater capacity for gene expression during exponential growth on fermentable carbon sources compared to during the diauxic shift and entry into the stationary phase [27].

It was possible to implement a very fine level of control over the timing and population density at which GFP expression was activated by varying glucose to sucrose ratios in the growth media. For example, with 0.5% glucose and 1.5% sucrose, GFP was fully induced after 5 hours of growth, at an OD_660nm_ of ~2 (Figure 2a). With 1% glucose and 1% sucrose the switch occurred after 9 hours, at an OD_660nm_ of ~6.4 (Figure 2b). When grown in medium containing 1.5% glucose and 0.5% sucrose, GFP expression was activated at 10 hours with a population density of ~7.3 (OD_660nm_), peaking after 11 hours at OD 8 (Figure 2c). With 2% glucose, and no sucrose in the growth medium, GFP expression was activated after 12 hours at an OD_660nm_ of ~9 (Figure 2d). These variations demonstrate the fine-tuning of gene expression afforded by this fully autonomous and cheap induction system.

**Figure 3:**
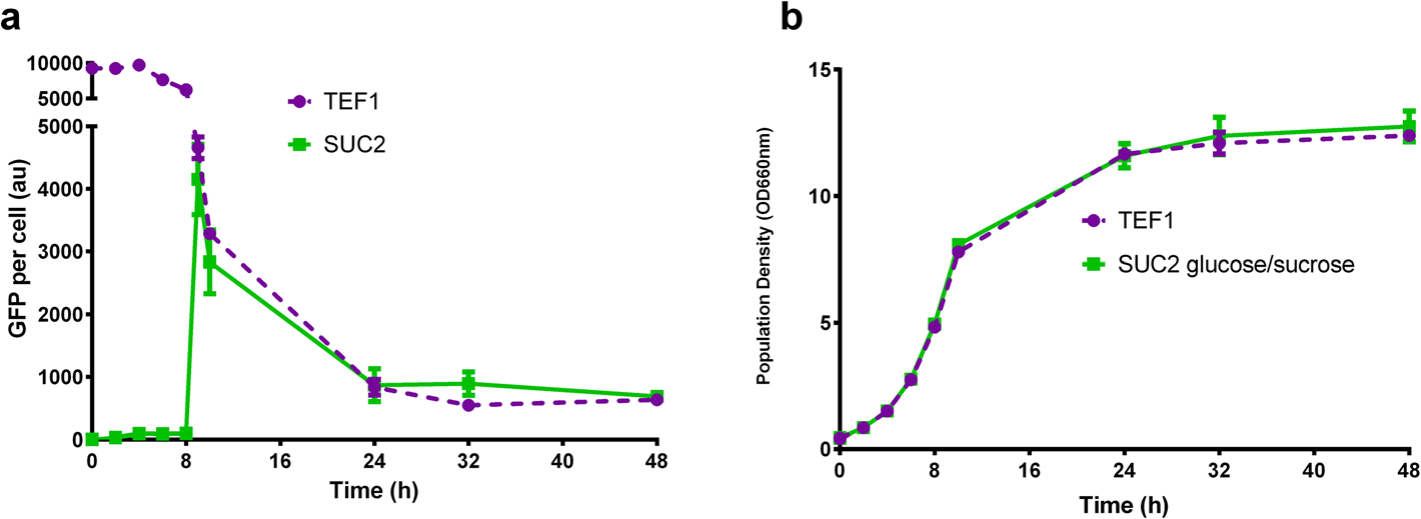
**Comparison of TEF1 and SUC2 promoter strengths.** GFP fluorescence **(a)** and population density **(b)** were measured for *P*_*TEF1*_-*GFP* and *P*_*SUC2*_-*GFP* expressing strains in 1% glucose, 1% sucrose containing medium over 48 hours. Mean and standard deviation for triplicate cultivations are shown.

For application to metabolic engineering scenarios, it is important that any dynamic regulatory system has a sustained level of induction throughout cultivation. Measurement of GFP expression levels over 56 hours of shake-flask cultivation showed that expression from the *SUC2* promoter remains at approximately 800–1000 fold higher than during the glucose consumption phase (Figure 2). This is a high expression level and after de-repression has occurred, is comparable to the commonly used *TEF1* promoter (Figure 3). As a point of reference, the *GAL10* promoter has a dynamic range of over 1000 [28] and an expression level similar to the *TEF1* promoter [29]. The *TEF1* promoter has previously been noted for its stable, high level of expression [29] and it was interesting that we observed a ~10 fold decrease in *TEF1* promoter mediated GFP expression between the early exponential phase and the diauxic shift (Figure 3). This difference could possibly be due to the fact that previous observations relied on the beta-galactosidase reporter gene, which has a half-life of around 20 hours in *S. cerevisiae* [30]. In contrast, our system utilised a destabilized version of the yEGFP gene that has a half-life of ~ 20 minutes [25], making it far more sensitive to any decreases in gene expression levels. The *TEF1* gene encodes a translational elongation factor that coordinates the positioning of aminoacylated tRNAs at ribosomes for the elongation of polypeptides [31]. Decreased expression from the *TEF1* promoter is consistent with the overall decrease in gene expression during and after the diauxic shift in *S. cerevisiae* [32,33].

### Testing sucrose mediated regulation of RNA interference

**Figure 4:**
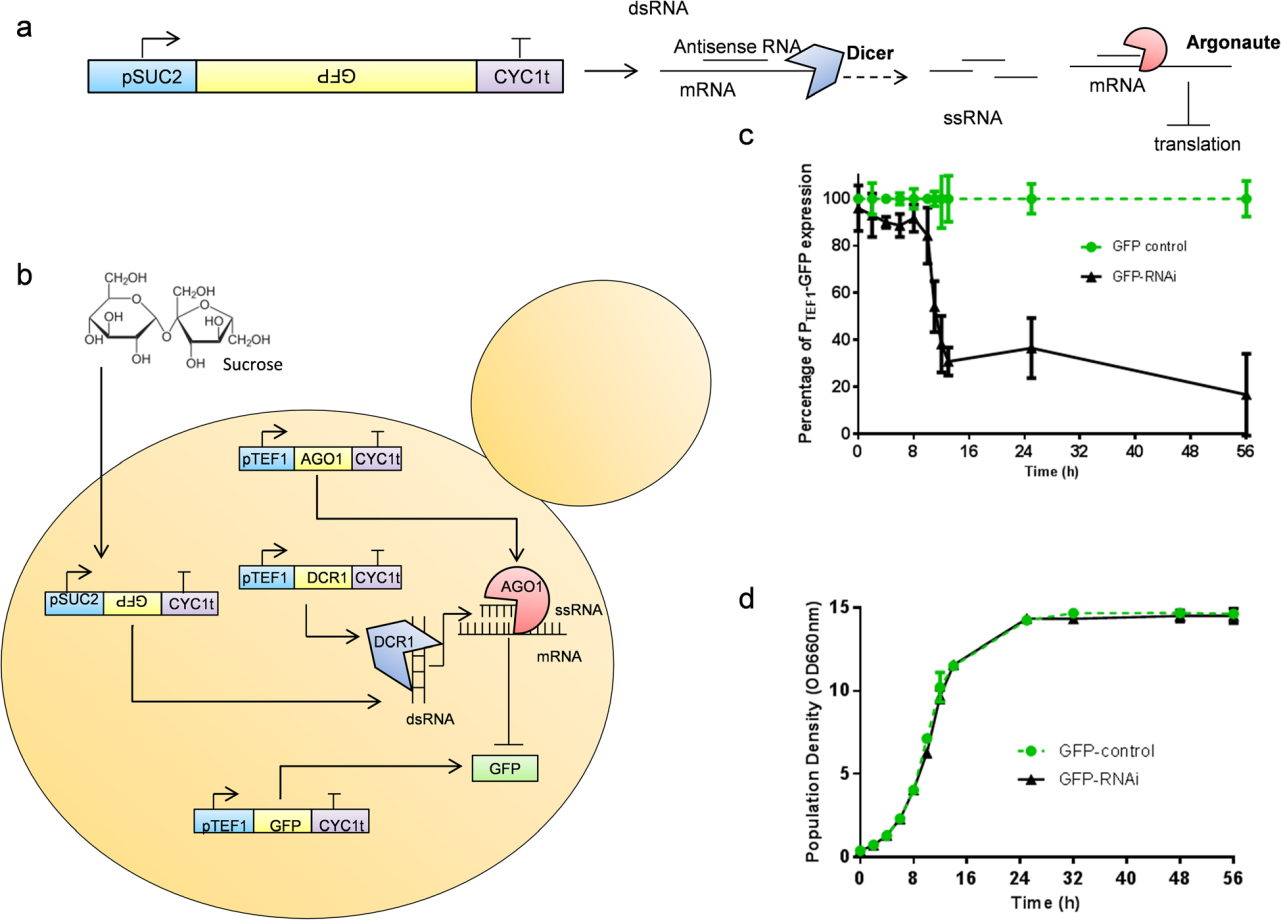
**Dynamic repression of GFP expression using sucrose mediated RNAi. (a)** The expression of an antisense RNA results in the destruction of complementary mRNA via Dicer and Argonaute enzymes. **(b)** Expression of the full GFP ORF in the antisense direction is triggered during growth on sucrose using the *SUC2* promoter, causing constitutively regulated (*TEF1* promoter) GFP expression to be repressed via Dicer/Argonaute-mediated RNA interference. GFP expression levels **(c)**, and population density **(d)** were measured for *P*_*TEF1*_-*GFP* expressing strains both with (‘RNAi’, black triangles) and without (‘control’, green circles) a *P*_*SUC2*_-*GFP* antisense construct. The GFP expression level from the control strain was set to ‘100%’, with GFP expression values from the RNAi strain being normalised to this value. Means ± standard deviations are shown from triplicate cultures. Figure 4b was adapted from [42]. Non-normalised GFP expression values are shown in Additional file 1: Figure S1.

Many of the most useful applications for dynamic regulatory systems require both the up-regulation and down-regulation of gene expression. To address this issue we sought to integrate the sucrose mediated induction system with a recently developed *S. cerevisiae* RNA interference (RNAi) system [5,34,35]. *S. cerevisiae* does not have a native system for RNA interference, but when the Argonaute and Dicer genes from *Saccharomyces castelii* are expressed, mRNAs can be specifically targeted for degradation by expressing complementary full-length antisense RNA [35] (Figure 4a), or double-stranded hairpin RNA [5,34,35].

Previous efforts at implementing dynamic control of RNAi resulted in significant leakiness of target-gene repression [5]. With the tight repression of expression from the *SUC2* promoter in the presence of glucose (Figure 2), we hypothesised that sucrose regulation would enable dynamic control of RNAi without non-induced repression occurring. The system was tested by expressing a destabilized GFP gene from the strong constitutive *TEF1* promoter, and targeting it for repression by expressing the entire GFP ORF in the antisense direction from the *SUC2* promoter in an RNAi capable strain (Argonaute and Dicer genes present) (Figure 4b). This strain was grown on medium containing a mixture of 1% glucose and 1% sucrose so that the *SUC2* promoter would activate GFP repression only after the glucose in the growth medium was consumed as previously demonstrated (Figure 2). GFP expression levels were normalised to a ‘control’ strain which was identical except for the absence of the GFP antisense construct that facilitates RNAi. During the glucose consumption phase (0–8 hr; Figure 2b) there was no significant difference between RNAi and control strains, suggesting that little or no non-specific repression was occurring. Between 11 and 13 hours, GFP levels dropped sharply, indicating rapid activation of the repression module, with fluorescence levels reduced to 30% of the control strain (Figure 4c). Repression was maintained with expression levels being further reduced to around 17% of the control strain by 56 hours. The two strains were cultivated in the same media and there was no difference in growth dynamics (Figure 4d).

## Conclusions

The dynamic control of gene expression is a core component of synthetic biology, and has great potential for the improvement of microbial cell factories. Dynamic control can be used to optimise a trade-off between growth based physiology and an organism’s engineered function [36]. By using dynamic regulation, any engineered functions that compete with normal growth-based physiology can be delayed until a population has reached a high density. Such functions are common, as even the most basic applications such as heterologous protein production can draw resources such as ATP, translation machinery, and amino acids away from native processes. In metabolic engineering the diversion of carbon flux, redox power, and ATP towards the production of target metabolites can reduce or potentially eliminate growth [1,2]. In support of this concept the *SUC2* promoter has previously been used for high-level expression of amylase enzymes [37,38].

Although purified sucrose is not commonly used as an industrial carbon source, it is often hydrolysed into its constituent monosaccharides glucose and fructose. It is possible that the use of the *SUC2* induction system would reduce the need for pre-hydrolysation of sucrose in industrial processes. For future directions it would also be interesting to test the sucrose induction system on an industrial carbon source such as sugarcane syrup at a much higher population density.

We present a simple dynamic regulatory system which does not require the addition of any expensive inducer compounds to the media, has a high dynamic range, low noise levels, maintains expression for prolonged periods, and can be coupled to RNAi to enable the repression of gene expression. We predict that this system will be of great utility for a diverse array of future applications in yeast cell factories.

Plasmids P_TEF1_-yEGFPCLN2PEST-pRS406, P_SUC2_-yEGFPCLN2PEST-pRS406, and P_SUC2_-GFPantisense-CYC1t-pRS413 will be made available from AddGene (www.addgene.org/).

## Materials and methods

### Media

Strain pre-cultures were grown in chemically defined liquid CBS medium with 5 g/L ammonium sulfate, 20 g/L glucose, vitamins and trace elements as described previously [39]. For GFP expression experiments CBS media with the indicated concentrations of glucose and/or sucrose was used. During strain construction purified amino acids (Sigma) were used to complement appropriate auxotrophies in agar plates (same composition as chemically defined media above) while YPD supplemented with appropriate antibiotics was used during gene deletion procedures. *E. coli* DH5α was used for plasmid propagation/storage and was grown in LB medium with kanamycin.

### Strains and plasmids

Primers, plasmids, and strains used in this study are shown in (Table 1, Table 2, and Table 3). respectively. Annotated vector maps and sequences can be found in ‘Additional file 2’. DNA manipulation and propagation were carried out using standard techniques [40] unless stated otherwise. All *S. cerevisiae* transformations were carried out using the lithium acetate method [41]. BAR1 and FUS1 genes were deleted in the base RNAi strain ‘S01’ as part of another related project which utilises pheromone quorum sensing for dynamic regulation (reference [42]), and are not important design features for this study. Strains transformed with yeast integrating plasmids were screened for correct single copy integration using PCR as previously described [43] except using primers LEU2A2 and LEU2D2 to screen to LEU2 locus.

**Table 1:** **Primers**

**Primer name**	**5′ to 3′ sequence**
MAL12F	TATTATctcgagACCAACCCGAAAATTCTTC
MAL12R	TATTATgaattcTTATGTAATTTAGTTACGCTTGAC
GPH1F	TATTATGatcgatTAGTTATCCGACTAGCAAG
GPH1R	TATTATgaattcTGTTCAAAATTAAATTAAGTTG
GLC3F	TATTATctcgagCGGTGATTTACAAGAAGAGG
GLC3R	TATTATatcgatTTTATTCTTGACGGTTCTTTATAC
SUC2F	TATTATctcgagACATACTAAGACATTTACCG
SUC2R	TATTATgaattcCATATACGTTAGTGAAAAGAAAAG
XhoI-pTEF1F	TATTATctcgagGCACACACCATAG
EcoRI-pTEF1R	TATTATgaattcTTGTAATTAAAACTTAGATTAGATTG
GFP1F	TATTATgaattcCTATATTACTTGGGTATTGCCC
GFP1R	TATTATcccgggTCTAAAGGTGAAGAATTATTCAC
LEU2A2	ATAGAATTGTGTAGAATTGCAG
LEU2D2	ATGAAATGAACATTGATTTACTATC
CYC1tF	TATAATtctagaACAGGCCCCTTTTCCTTTGT
CYC1tR	TATTATgagctcACGATGAGAGTGTAAACTGC
ApaI-SUC2F	TATTATgggcccACATACTAAGACATTTACCG
ClaI-SUC2R	TATTATatcgatCATATACGTTAGTGAAAAGAAAAG

Restriction enzyme sites are shown in lower case.

**Table 2:** **Plasmids**

**Name**	**Details**	**Origin**
pRS406	URA3 integrating vector	[45], Euroscarf
pSF019	TEF1 promoter containing vector	[29]
yEGFPCLN2PEST-pRS406	Destabilized GFP base plasmid	[36]
P_MAL12_- yEGFPCLN2PEST-pRS406	MAL12 promoter driven GFP expression	This study
P_GLC3_- yEGFPCLN2PEST-pRS406	GLC3 promoter driven GFP expression	This study
P_GPH1_- yEGFPCLN2PEST-pRS406	GPH1 promoter driven GFP expression	This study
P_SUC2_- yEGFPCLN2PEST-pRS406	SUC2 promoter driven GFP expression	This study
P_TEF1_-yEGFPCLN2PEST-406	Constitutive TEF1 promoter driven GFP expression	This study
pRS413	Yeast centromeric plasmid with HIS3 selection marker	[45], Euroscarf
CYC1t-pRS413	CYC1 terminator	This study
P_SUC2_-CYC1t-pRS413	SUC2 promoter, CYC1 terminator	This study
P_SUC2_-GFPantisense-CYC1t-pRS413	SUC2 regulated expression of an antisense RNAi construct for GFP	This study
pRS404-PTEF -Ago1-CYC1t	TRP1 integrating vector with constitutive Argonaute expression	[5]
pRS405-PTEF-Dcr1-CYC1t	LEU2 integrating vector with constitutive Dicer expression	[5]
pUG6	LoxP-KanMX-LoxP cassette	Euroscarf
pUG66	LoxP-Ble-LoxP cassette	Euroscarf

**Table 3:** ***S. cerevisiae*** **strains used in this study**

**Name**	**Genotype**	**Notes**	**Origin**
CEN.PK113-5D	MATa; ura3-52; MAL2-8^C^; SUC2	Haploid MATa lab strain with uracil auxotrophy	Euroscarf
CEN.PK113-7D	MATa; MAL2-8^C^; SUC2	Prototrophic haploid MATa lab strain	Euroscarf
S01	CEN.PK2-1c, bar1Δ	*BAR1* gene deleted	This study
S02	CEN.PK2-1c, bar1Δ, fus1::KanMX, trp1::pRS404-PTEF-Ago1	*BAR1* and *FUS1* deleted, Argonaute gene integrated at *TRP1*	This study
S03	CEN.PK2-1c, bar1Δ, fus1::KanMX, trp1::pRS404-PTEF-Ago1, leu2::pRS405-PTEF-Dcr1	RNAi capable base strain with Argonaute and Dicer integration	This study
GFP01	CEN.PK113-5D, bar1::phleo, fus1::KanMX, ura3-52::P_TEF1_-GFPCLN2PEST-ADH1t-pRS406	constitutive destabilized GFP expression	This study
GFP02	CEN.PK113-5D, ura3::P_MAL12_-GFPCLN2PEST-ADH1t-pRS406	*MAL12* regulated GFP expression	This study
GFP03	CEN.PK113-5D, ura3::P_GLC3_-GFPCLN2PEST-ADH1t-pRS406	*GLC3* regulated GFP expression	This study
GFP04	CEN.PK113-5D, ura3::P_GPH1_-GFPCLN2PEST-ADH1t-pRS406	*GPH1* regulated GFP expression	This study
GFP05	CEN.PK113-5D, ura3::P_SUC2_-GFPCLN2PEST-ADH1t-pRS406	*SUC2* regulated GFP expression	This study
GFP06	CEN.PK2-1c, bar1Δ, fus1::KanMX, ura3:: P_TEF1_-yEGFPCLN2PEST-406, trp1::pRS404-PTEF-Ago1, leu2::pRS405-PTEF-Dcr1, pRS413	RNAi capable, constitutive GFP expressing control strain	This study
GFP07	CEN.PK2-1c, bar1Δ, fus1::KanMX, ura3:: P_TEF1_-yEGFPCLN2PEST-406, trp1::pRS404-PTEF-Ago1, leu2::pRS405-PTEF-Dcr1, P_SUC2_-GFPantisense-CYC1t-pRS413	*SUC2* promoter mediated repression of GFP using RNAi	This study

All putative sucrose-responsive promoters were PCR amplified from CENP.K2-1c genomic DNA, and contain ~700 bp upstream of the start codon of the native gene. *MAL12* (XhoI/EcoRI), *GPH1* (ClaI/EcoRI), *GLC3* (XhoI/ClaI), and *SUC2* (XhoI/EcoRI) promoter regions were cloned 5’ of the start codon in the GFPCLN2PEST-pRS406 plasmid using the indicated restriction enzyme combinations. The P_TEF1_-yEGFPCLN2PEST-pRS406 plasmid was made by inserting the *TEF1* promoter amplified from pSF019 using primers 9 and 10 into the yEGFPCLN2PEST-pRS406 plasmid with XhoI/EcoRI.

The GFP silencing construct was expressed using the *SUC2* promoter on the low copy number pRS413 plasmid. To construct this expression cassette a *CYC1* terminator region was PCR amplified from genomic DNA using CYC1tF and CYC1tR primers and cloned into pRS413 using XbaI and SacI. The *SUC2* promoter was then amplified using ApaI-SUC2F and ClaI-SUC2R and inserted 5’ of *CYC1t* in pRS413 to create P_SUC2_-CYC1t-pRS413. The GFP silencing construct was made by cloning the full length GFPCLN2PEST ORF sequence in the antisense direction between the *SUC2* promoter and *CYC1* terminator in P_SUC2_-CYC1t-pRS413 using GFP1F/GFPR primers and EcoRI/XmaI restriction enzymes to create the P_SUC2_-GFP1i-CYC1t-pRS413 plasmid.

### Fermentation conditions

All growth experiments were carried out in baffled screw top flasks shaking at 200 rpm, 30°C with medium comprising 10% of the total flask volume. Individual colonies were transferred to liquid media and pre-cultured for approximately 15 hours. Cultures were then passaged into a second pre-culture and grown to mid exponential phase (OD_660nm_ between 1 and 6) prior to inoculation of experimental cultures at an OD_660nm_ of 0.1 for the initial promoter comparison (Figure 1) and 0.4 for the time course experiments (Figure 2).

### Analytics

GFP fluorescence was measured using an Accuri C6 flow cytometer (BD Biosciences) as in [36]. HPLC was used to measure sucrose, fructose, and glucose as previously described [44].

### Statistical analysis

Mean GFP fluorescence values from triplicate experiments were compared using a two-sided, unpaired student’s *t*-test with equal variance. Null hypotheses were rejected when p-values were ≤ 0.05.

## Additional files

Additional file 1: Figure S1. Supplementary material.

Additional file 2: **Annotated plasmid maps and sequences.**
