# Supplementary material for: Dynamic regulation of gene expression using sucrose responsive promoters and RNA interference in Saccharomyces cerevisiae
Source: Microb Cell Fact. 2015 Apr 1;14:43. doi: 10.1186/s12934-015-0223-7 (PMC4427958; doi:10.1186/s12934-015-0223-7)
Supplement: Supplementary file 1 — Supplementary material. [file 12934_2015_223_MOESM1_ESM.docx]

Supplementary Information for:

# Dynamic Regulation of Gene Expression Using Sucrose Responsive Promoters and RNA interference in *Saccharomyces cerevisiae*

Thomas C. Williams, Monica I. Espinosa, Lars K. Nielsen, Claudia E. Vickers^§^

Australian Institute for Bioengineering and Nanotechnology (AIBN)

The University of Queensland, St. Lucia, QLD 4072, Australia

^§^Corresponding author

Email addresses:

TCW: [t.williams1@uq.edu.au](mailto:t.williams1@uq.edu.au)

MIE: [monica.espinosa@uq.net.au](mailto:monica.espinosa@uq.net.au)

LKN: [lars.nielsen@uq.edu.au](mailto:lars.nielsen@uq.edu.au)

CEV: [c.vickers.@uq.edu.au](mailto:c.vickers.@uq.edu.au)


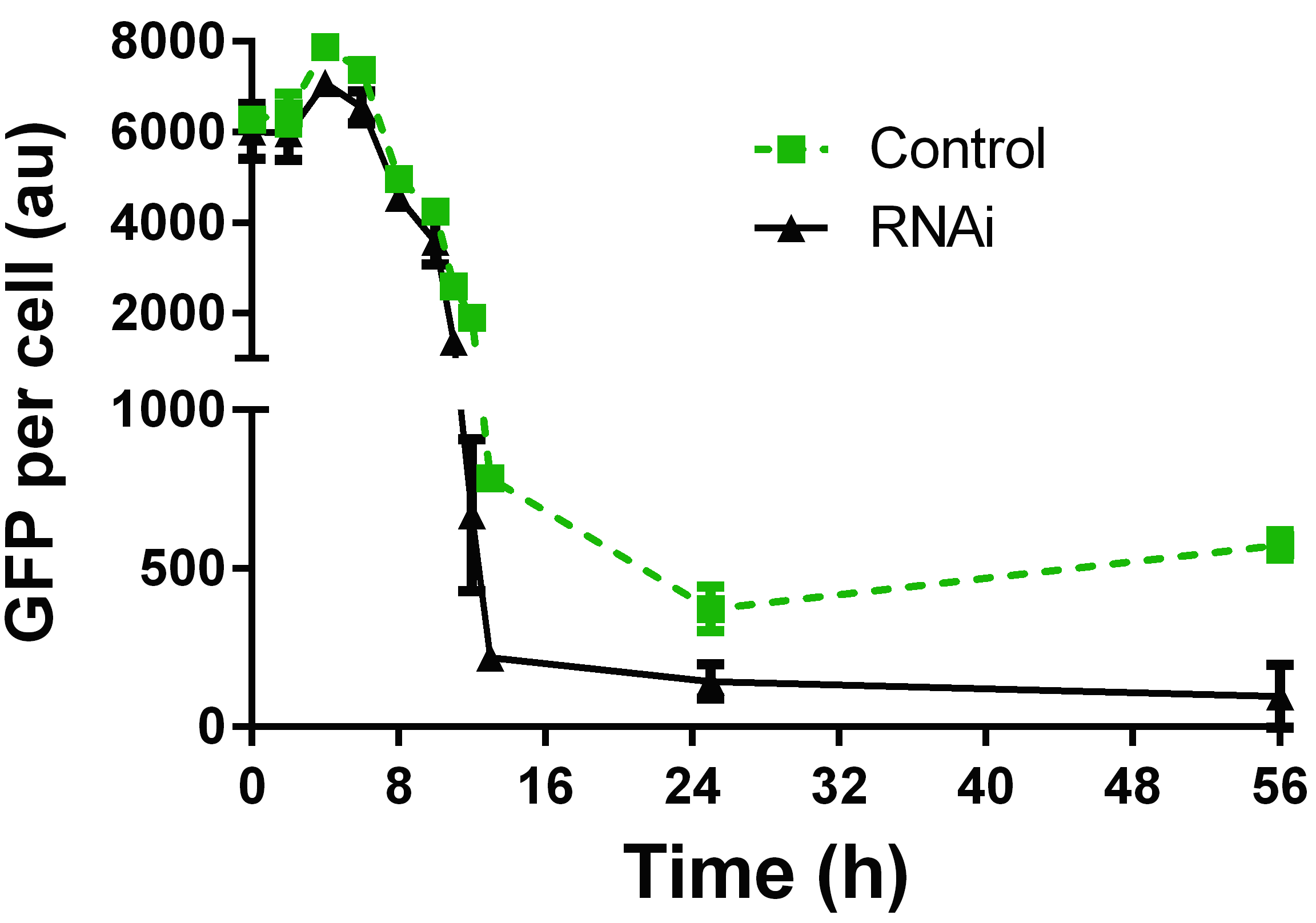


Supplementary Figure 1. RNAi knockdown of TEF1-GFP expression using the SUC2 promoter.

GFP expression levels were measured for *P_TEF1_*-*GFP* expressing strains both with (‘RNAi’, black triangles) and without (‘control’, green squares) a *P_SUC2_*-*GFP* antisense construct. Absolute expression vales are shown throughout the course of a fermentation.
